# Using music to assist language learning in autistic children with minimal verbal language: The MAP feasibility RCT

**DOI:** 10.1177/13623613241233804

**Published:** 2024-03-03

**Authors:** Tim I Williams, Tom Loucas, Jacqueline Sin, Mirjana Jeremic, Sina Meyer, Sam Boseley, Sara Fincham-Majumdar, Georgia Aslett, Ruan Renshaw, Fang Liu

**Affiliations:** 1 University of Reading, UK; 2 City, University of London, UK

**Keywords:** autism, early language learning, music, parent–child interaction, social communication

## Abstract

Music has been shown to improve social interaction and attention to verbal stimuli in autism. We report a feasibility randomised controlled trial of an online intervention using music-assisted programmes, compared with best-practice treatment (Social Communication Intervention for Pre-schoolers–Intensive) for language learning in preschool autistic children with minimal verbal language. Minimisation randomisation ensured comparability of groups before intervention. Ninety-one people expressed interest in taking part; 27 met eligibility criteria and were randomised to receive either music-assisted programmes or Social Communication Intervention for Pre-schoolers–Intensive. Children and their parent received two 45-min sessions weekly, over 18 weeks, coached online by a speech and language therapist. A smartphone app was developed to support home-based practice between sessions. Over the study period, 20% of participants completed the intervention and assessments of outcome measures. At 3 months post-intervention follow-up, social responsiveness, understanding of words and phrases and number of words spoken and parent–child interaction improved more in the music-assisted programmes than the Social Communication Intervention for Pre-schoolers–Intensive group. The results demonstrate the feasibility of recruiting this population into a randomised controlled trial and the music-assisted programmes had high perceived acceptability highlighted by parent interviews. A full clinical trial to establish music-assisted programmes’ effectiveness in improving early vocabulary learning in autistic children is warranted.

**Lay abstract:**

Research has shown that autistic individuals often have unusually good musical skills and that combining words and music helps autistic individuals to focus on spoken words. This study tests the idea that music will help with early language learning of preschool autistic children. The results show that when caregivers sing words to autistic children, the children pay more attention to the caregiver than when the words are spoken and that they learn word combinations more easily.

## Introduction

Autism spectrum disorder (autism hereafter) is characterised by the early onset of differences in social communication and social interaction, and restricted, repetitive patterns of behaviour (American Psychiatric Association, 2013). About 30% of autistic children do not develop functional language despite extensive interventions (Hampton & Kaiser, 2016; Oono et al., 2013; Tager-Flusberg & Kasari, 2013). Children who do not develop functional language by 5 years of age have poorer outcomes in adulthood (Billstedt et al., 2005; Howlin et al., 2004). Limited language skills are also associated with behaviour that challenges (Chiang, 2008) and poorer quality of life outcomes (García-Villamisar & Dattilo, 2010) with increased support needs from families (Bozkurt et al., 2019; Emily & Grace, 2015). Early post-diagnosis intervention is beneficial (Volkmar, 2014), and delays in early intervention are associated with increased caregiver stress and can jeopardise long-term positive outcomes (Dimian et al., 2021; Zwaigenbaum et al., 2015). Some studies have shown that starting interventions before age 5 resulted in significant gains in cognition and adaptive behaviour (Baghdadli et al., 2007; Billstedt et al., 2005; Magiati et al., 2014). Improving functional communication early is therefore an important target for interventions (Frazier et al., 2021).

There are five key phases of spoken-language development for autistic children, including preverbal communication, first words, word combinations, sentences and complex language (Tager-Flusberg et al., 2009). Existing programmes have not created meaningful change on spoken-language outcomes in autistic children, with improvement limited to using single words (Hampton & Kaiser, 2016). A Cochrane review of communication interventions for autistic children with little or no language found no evidence of effectiveness on functional language (Brignell et al., 2018). A wide range of interventions have also failed to improve functional communication skills in autism, including traditional therapy (Sandiford et al., 2013), speech repetition therapy (Chenausky et al., 2016), relational music therapy (Gattino et al., 2011), auditory integration therapy and other sound therapies (Sinha et al., 2011), intonation- or melody-based training programmes (Chenausky et al., 2016; Lim, 2010; Sandiford et al., 2013), augmentative and alternative communication interventions (Flippin et al., 2010; Lorah et al., 2015) and various other approaches addressing social, communicative and cognitive skills in autism (Prelock & Nelson, 2012). Thus, there remains a need for intervention that can support the development of functional spoken language in autistic children with no or few words.

Music may assist language learning in autism in three ways. First, autistic children prefer musical stimuli over speech (Blackstock, 1978), and music may increase their orientation to speech (Janzen & Thaut, 2018). Neuroimaging studies indicate that music enhances attention to language in autistic children (Lai et al., 2012; Sharda et al., 2015). Lai et al. (2012) found increased connectivity and greater activation of the left inferior frontal gyrus to song than speech in autistic compared with non-autistic children. Sharda et al. (2015) similarly found improved frontotemporal connectivity during sung-word listening in comparison to spoken-word listening. Since language and communication difficulties in autism are associated with reduced streamlines in the left arcuate fasciculus along the frontotemporal pathway (Catani et al., 2016), music-based language interventions may induce structural and functional changes in the autistic brain to facilitate language learning.

Second, autism has been associated with enhanced musical processing (O’Connor, 2012; Ouimet et al., 2012), including pitch discrimination and memory (Heaton et al., 2008), pitch recognition (Mottron et al., 1999) and identification of musical chords (Heaton, 2003) and melodic contour (Jiang et al., 2015). There is some evidence that the enhanced musical skills are restricted to individuals with stronger autistic traits (Altgassen et al., 2005; Jones et al., 2009). Furthermore, autistic individuals are able to identify emotions in music (Molnar-Szakacs & Heaton, 2012; Quintin, 2019), and social communication in autism can be improved through music (Sharda et al., 2018). Given that autistic individuals show an ability to perceive and imitate complex sound sequences including speech intonation and musical melody (Wang et al., 2021, 2022), learning language through singing may produce promising results.

Third, reviews of music therapy suggest that music improves behaviour, social communication, brain connectivity, quality of life and parent–child relationships (Gassner et al., 2022; Geretsegger et al., 2022; James et al., 2015). In particular, based on a large randomised controlled trial (RCT) involving 364 children aged 4–7 across nine countries (Bieleninik et al., 2017), while improvisational music therapy might not increase social affect as assessed by the Autism Diagnostic Observation Schedule (Gotham et al., 2006), it was associated with improved social motivation and awareness and autistic mannerisms as reported by parents (Constantino & Gruber, 2012) compared to enhanced standard care. Music interventions can take two forms: (1) receptive, where the therapist presents musical stimuli, and (2) active, where the child takes part in the music making (Geretsegger et al., 2015; Wigram & Gold, 2006). James et al. (2015) found that both receptive and active approaches produced similar results, while Geretsegger et al. (2022) estimated that receptive techniques had smaller effects than active methods. Music therapy is found to be more useful when combining improvisation and pre-recomposed music and activities (Wheeler et al., 2005). Autistic children show increased spontaneous imitation and social reciprocity in child-led musical play routines (Stephens, 2008). Similarly, music therapy involving improvised music making facilitates family interactions (Oldfield et al., 2012), and there is some evidence that prescriptive songs may promote acquisition of social skills for autistic children (Pasiali, 2004). Thus, we designed a blended approach of receptive and improvisational music methods to support the development of functional spoken language in autism.

Many music therapy studies concentrate on the therapist as intervention agent; however, in autism it is common for therapists to train parents to implement the intervention (Oldfield et al., 2012; Pasiali, 2004). Across interventions, parent delivery appears to maximise benefits (Parsons et al., 2017; Soorya et al., 2015). For instance, Hampton and Kaiser (2016) suggest that children gained more functional language and there was generalisation of newly acquired skills across environments when parents were involved (Krasny et al., 2003; Williams White et al., 2007). However, systematic reviews of music therapy refer exclusively to therapists intervening directly with the child (Gassner et al., 2022; Geretsegger et al., 2022; James et al., 2015). We planned to deliver an in-person clinic-based intervention, but COVID-19 restrictions on face-to-face work led to redesigning delivery as a tele-intervention to minimise infection risks. Telehealth interventions for autistic individuals have been established as equivalent to face-to-face interventions, with reduced costs and high parent satisfaction (Sutherland et al., 2018). A recent systematic review provides initial evidence that parent-mediated telehealth interventions improve social communication in autistic children who live outside of urban areas, with increased parent knowledge and parent intervention fidelity (Parsons et al., 2017). Thus, in our trial, the therapist modelled for and instructed the parents on intervention techniques through live online videoconferencing. In parallel, a smartphone app was developed to provide parents with modelled songs to support homework practice between sessions.

Finally, in keeping with the ‘*Lancet* Commission on the future of care and clinical research in autism’ (Lord et al., 2022), we included a control arm that received a best-practice communication intervention as recommended by the National Institute for Health and Clinical Excellence (NICE, 2013) guideline. Social Communication Intervention for Pre-schoolers–Intensive (SCIP-I) is a therapy programme developed at the University of Reading Speech and Language Therapy Paediatric Clinic that aims to develop children’s social communication and interaction using a parent-mediated approach (Loucas & Fincham-Majumdar, 2019).

The aim of this study was to demonstrate the feasibility of running an RCT comparing a music-based language intervention to a best-practice communication intervention in young autistic children (Williams et al., 2021). The objectives of this trial were (1) to determine the feasibility of carrying out an RCT comparing the music-assisted programmes (MAP) with a best-practice communication intervention (SCIP-I) delivered remotely to preschool autistic children with little or no spoken language, (2) to provide data to estimate sample size and effect size for a full-scale trial, (3) to optimise the music-assisted intervention through a post-intervention interview study with parents and (4) to pilot and refine an app available on smartphones for supporting and recording home-practice sessions alongside the intervention.

## Methods

### Study design

The study used an RCT comparing a best-practice speech and language intervention (SCIP-I) with the MAP for learning 36 target words (Table 1). In the SCIP-I group, sessions were given focusing on social communication strategies with focused stimulation of the target words. In the MAP group, a structured training method was delivered through naturalistic, interactive activities using songs to teach the target words. The target words were chosen based on the following: appropriateness to the children’s age range based on the published literature; questionnaires to parents asking which words parents would like their children to learn and consideration of which words were most functional for the children. Parents were given access to the initial list of target words (top seven rows, Table 1). When a word (e.g. ‘hungry’) already spoken by the child was identified, a word of the same category (e.g. ‘thirsty’) from the backup list (bottom seven rows, Table 1) would be chosen as the replacement. Among these target words, ‘singing’ (or ‘sleeping’) rather than ‘sing’ (or ‘sleep’) was used because it was part of the lyrics in the songs, for example ‘Singing, dancing and we’ll play’ (or ‘Time for sleeping’).

**Table 1.** The list of 36 target words for the child to learn during the trial, and the list of 35 backup words for replacement of those already known by the child.

Thirty-six target words					
mummy	daddy	hands	home	toys	book
TV	park	ball	ouch	cat	bye-bye
bed	food	red	green	happy	sad
singing	stop	go	kiss	play	sleeping
hello	help	drink	look	tired	hungry
yes	no	more	please	yummy	thank you
Thirty-five backup words					
bump	rainbow	sky	bubbles	belly	music
car	dog	blue	yellow	finished	rub
in	outside	bath-time	night-time	clap	driving
sitting	smiling	yay	brum	dancing	hug
fall	grandma	sister	brother	grandpa	cold
thirsty	hot	feet	teddy	water	

### Participants and settings

A sample size of 30 autistic children was considered adequate for determining feasibility on the grounds of comparability to sample sizes in similarly designed published studies, which ranged from 6 (Wan et al., 2011), through 12 (Sandiford et al., 2013) and 23 (Chenausky et al., 2016), to 50 divided across three groups (18, 18, 14 in each group; Lim, 2010). The intended sample size was also established according to the precision required for the critical objectives (e.g. estimation of recruitment rate), rather than through power analysis (Arain et al., 2010).

The study recruited children aged between 24 and 60 months with a clinical diagnosis of autism, with little or no spoken language defined operationally as having fewer than 20 words used for functional communication (Kasari et al., 2013). The autism diagnosis was further confirmed using the recommended cut-off scores on the Social Responsiveness Scale–2 (SRS-2; Constantino & Gruber, 2012; Moody et al., 2017). Participants were recruited between September 2019 and August 2021 from special education preschool provision, National Health Service (NHS – state funded), voluntary groups, social media and privately funded clinics in the United Kingdom. Potential participants completed an online questionnaire assessing the child’s eligibility. Exclusion criteria were (adapted from Dawson et al., 2010, p. e18) as follows:

A neurodevelopmental condition of known aetiology (e.g., fragile X syndrome);

Significant hearing or motor condition;

Major physical problems such as a chronic serious health condition;

Seizures at time of entry;

History of a serious head injury and/or neurologic disease;

IQ below 50 as measured by the Vineland Adaptive Behavior Scales – Third Edition (Sparrow et al., 2016);

Not meeting the cut-off score for autism on the SRS-2 (Constantino & Gruber, 2012).

Interventions and assessments took place via video link or telephone with the participant and their parent being at home and the therapist and assessor being either at the university or in their own home.

### Ethics

Ethical approval was granted by the funder’s (European Research Council) ethics review panel, the NHS (National Health Service) HRA (Health Research Authority) and HCRW (Health and Care Research Wales) Approval service, and the University of Reading research ethics committee. Since the children were unable to give informed assent, written informed consent was sought from parents. Children’s assent was monitored by their behaviour and responses to their parents during the sessions.

### Community involvement

There was no official community involvement in the present study. However, a focus group study was conducted before the trial with parents of autistic children gathering their views about their children’s language learning needs and experiences. We also sought parents’ input about the words they wished their children to learn via an online questionnaire before the study. A convenience sample subset of the MAP group parents was interviewed post-intervention to gather their views and experiences of the trial.

### Interventions

UK COVID-19 regulations required social distancing which was achieved by asking parents to carry out all assessments and interventions with live guidance from the research speech and language therapist (M.J.) using Microsoft Teams. Both interventions consisted of 36 therapist-led training sessions, lasting 45 min, twice a week for 18 weeks.

In each session, the parent was coached by the therapist to carry out a range of activities with the child. In addition, parents were encouraged to implement a minimum of five 10-min practice sessions with their children per week. Each week, one session was recorded and jointly reviewed by the therapist and the parent to encourage the parent to reflect on their interactions and enable better tailoring of the treatment. Parents of the MAP group were provided with an Android app (the MAP app hereafter), which was specifically developed to support and record home practice by tracking the number and duration of practice sessions undertaken by each parent–child dyad.

The MAP intervention was as follows (Williams et al., 2021): (1) At the beginning of the intervention, a Hello song and a Bye-bye song were introduced using the MAP app. Thereafter, a new song with a common theme such as colours, play, food or emotions was introduced every week or every couple of weeks. There were 11 songs in total, featuring 36 target words which were paired with pictures (see Supplementary Table 1). Once a song was introduced, the parents could play and sing the song together with the child as often as they liked. (2) All sessions started with the Hello song and ended with the Bye-bye song. During each session, the songs were played using a computer or a smartphone and sung with a range of home-made music instruments such as shakers. The children were taught to sing the songs, in which the target words occurred repeatedly, together with other engaging and interactive activities such as dancing, vocalising, improvising and playing musical games. (3) Naturalistic teaching strategies were used, such as incidental learning, high-density repetition, time delay and mand-modelling. Parents were taught strategies including intensive interaction and communication temptations to help them engage with their children.

For the SCIP-I group, intervention was modelled on that provided in usual clinical practice for young children with little or no spoken language in the University of Reading Speech and Language Therapy Paediatric Clinic but delivered via online methods. It was based on the SCIP (Loucas & Fincham-Majumdar, 2019) modified to teach the 36 target words identified. SCIP-I is a naturalistic developmental intervention for the core features of autism and uses a parent-mediated approach as recommended by the NICE guideline (National Institute for Health and Clinical Excellence, 2013). The SCIP-I group participants were also asked to complete five sessions of 10-min home practice each week.

### Randomisation

We used the free open-source web-based Python programme, MinimPy (Saghaei & Saghaei, 2011), to allocate participants randomly to either the MAP or SCIP-I group. Using minimisation randomisation, the imbalance in prognostic factors of gender, developmental level (Vineland developmental quotient ⩾70 or 50–69) and echolalia (present or absent, as reported by the parents) was controlled for between groups (Taves, 2010). Participant allocation was adequately concealed before assignment. The Python programme was run (by F.L., who was only provided with a de-identified participant code, gender, developmental level and echolalia status) each time a participant enrolled to provide an assignment as they were recruited.

### Outcomes and measures

In accordance with government regulations regarding social contact during the COVID-19 epidemic, all measures were administered by parents at home while being coached by the research speech and language therapist (M.J.) over audio or video.

#### Feasibility measures

Measures of feasibility included participant recruitment rate, screening for eligibility, parents’ acceptance to randomisation allocation, session attendance, retention to follow-up and acceptability of intervention.

#### Outcome measures

The following outcome measures were collected across four timepoints: pre-intervention, mid-intervention (12 weeks into the intervention), post-intervention and 3-month follow-up (see Supplementary Table 2 for details).

1. The number of target words understood and produced out of the total of 36 (Table 1) by the child was assessed. Comprehension was measured by the therapist presenting four pictures at a time using PowerPoint and asking the child to point to the picture representing the target word. In the production task, the pictures of the 36 target words were presented one at a time in a pseudorandomised order using PowerPoint, and the child was asked to name the pictures.
2. Participants’ social responsiveness was assessed using the SRS-2 (Constantino & Gruber, 2012). The SRS-2 is a questionnaire that uses a scale of 1–4 (1 = not true, 4 = almost always true) to rate items, with higher scores indicating less social responsiveness (Constantino et al., 2000, 2004). A designated raw score cut-off value of 70 is considered to have a sensitivity of 0.78 and specificity of 0.94 for autism (Constantino & Gruber, 2005). Raw total scores are converted to gender-normed *T* scores, with a *T* score of 75 indicating reduced social responsiveness.
3. Expressive and receptive vocabulary skills were measured using the Expressive One-Word Picture Vocabulary Test–4 (EOWPVT-4; Martin & Brownell, 2010) and the Receptive One-Word Picture Vocabulary Test–4 (ROWPVT-4; Martin & Brownell, 2011). The EOWPVT-4 and ROWPVT-4 tests provide raw scores which can be used to determine standard scores, age-equivalent scores and percentile ranks. The tests are appropriate for use with typical and atypical populations, including those with learning difficulties.
4. The number of words and phrases understood and the number of words produced by the child were collected through parent report using the MacArthur-Bates Communicative Development Inventories (CDIs): Words and Gestures Forms (Fenson et al., 2007). The CDI has high caregiver–teacher agreement for autistic children (intra-class correlation 95% confidence interval estimates 0.77–0.93; Nordahl-Hansen et al., 2013).
5. Social communication was evaluated from a 10-min sample of free play between the child and their parent recorded by the parent at home. Following Meirsschaut, Warreyn and Roeyers (2011), codes for social responses were defined as verbal and non-verbal behaviours as a response to someone else’s initiation, including responding to name, following a command, responding to joint attention or other social interaction and repeating someone’s behaviour. Codes for social initiations were defined as verbal and non-verbal attempts to interact with another person, including requesting, showing, giving, pointing, commenting, vocalising and maintaining (active involvement to keep a play going; Meirsschaut et al., 2011). Coding was done by authors S.M. and S.B. The intra-class correlation for social initiations was 0.902 and 0.873 for social responses, signifying excellent inter-rater reliability (Hallgren, 2012).
6. Functional language was also measured from the 10-min parent–child interaction session. Three categories were coded, including vocalisations, words and phrases (Tager-Flusberg et al., 2009). Vocalisations included any type of canonical or non-canonical babbling such as ‘mamama’. Words included spoken or sung words and word approximations that were recognisable or recognised by parents, such as ‘hat’ and ‘mahkey’ (monkey). Phrases included spoken or sung phrases as word combinations, such as ‘let’s go’ and ‘knees and toes’. Coding was done by authors S.M. and S.B. Intra-class correlation showed excellent inter-rater reliability for vocalisations (0.901) and phrases (0.967) and a fair inter-rater reliability for words (0.540).
7. Communication, daily living skills, socialisation and maladaptive behaviour were measured using the comprehensive parent/caregiver form of Vineland Adaptive Behaviour Scales–third edition (VABS-3; Sparrow et al., 2016), filled out by parents. The VABS-3 has high internal consistency (Cronbach’s α range 0.86–0.97), good test–retest reliability (corrected *r* values range 0.62–0.92) and good inter-rater reliability (range 0.61–0.87).

### Perceived acceptability

To establish the perceived acceptability of MAP intervention, following the intervention completion, a convenience sample of MAP group parents was invited to a semi-structured interview about their experience of receiving the MAP intervention, including use of teletherapy, parent-mediated approach, intensity and duration of intervention, the songs used and the usability of the MAP app. Parents were also asked about their views on the outcome measures used and if and how they found MAP helped their child.

### Analysis plan

The analysis was designed to provide data for calculating sample size and trial parameters in future studies. Feasibility data were analysed using descriptive statistics. Outcome data were analysed using linear mixed-effects models in R (R Core Team, 2023), with packages of *lme4* (Bates et al., 2015) and *lmerTest* (Kuznetsova et al., 2017). Group (effect-coded: MAP vs SCIP-I) and timepoints (effect-coded: pre-, mid-, post-intervention, 3-month follow-up) were included as fixed effects and participants as random effects using Type III analysis of variance table with Satterthwaite’s method. Post hoc analyses of the interaction effects were investigated with the *emmeans* package with *p* values adjusted using the Holm’s method (Lenth et al., 2020). Interviews were transcribed verbatim and analysed using thematic analysis (Braun & Clarke, 2006). Codes were checked and cross-referenced by S.F.-M. and T.L.

## Results

### Feasibility data

**Figure 1.**
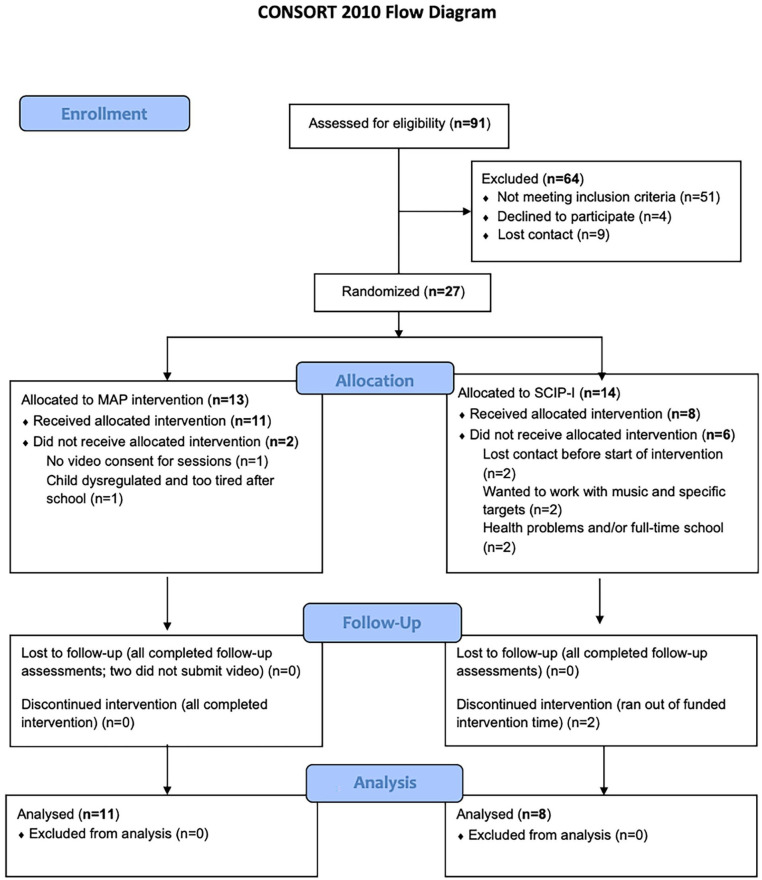
CONSORT flow diagram of recruitment rates to MAP feasibility trial. Ninety-one people expressed interest in taking part in the trial of those only 27 could be randomised. Once randomised a total of eight withdrawals were recorded. The difference in withdrawal rates between conditions was not significant (Fisher’s exact test χ^2^ = 0.21).

Figure 1 shows the CONSORT flow diagram (see Supplementary Table 3 for details), and Table 2 displays recruitment and assessment rates of the trial. Between the study recruitment period (1 September 2019–31 August 2021), a total of 91 people expressed interest in taking part in the trial (65 filled in the online survey form; 26 contacted the team by email or telephone). Of these, 64 were screened, and 29 completed all the eligibility assessments. Twenty-seven completed baseline assessments and were randomly assigned, with 13 to the MAP arm and 14 to the SCIP-I. The most common reasons for not meeting eligibility criteria were (1) no formal diagnosis of autism, (2) more than 20 spoken words albeit many of these children were not using them functionally and (3) more than 5 years old.

**Table 2.** Recruitment and assessment rates of the trial.

Number contacted to take part = 91
Number interviewed = 64
Number completing initial assessments = 29
Number taken into trial = 27
Number completing the intervention = 19
Number assessed at 3 months post trial = 19
Number of assessments completed at each time point = all but two participants completed all the assessments (for one SCIP-I and one MAP participant data are incomplete at 3 months post trial)
Number of sessions completed = all but two SCIP-I participants completed all the sessions

SCIP-I: Social Communication Intervention for Pre-schoolers–Intensive; MAP: music-assisted programmes.

Throughout the study duration (1 September 2019–28 February 2022), eight participants (29.6% of 27 randomised), of whom six had been allocated to SCIP-I and two allocated to MAP, disengaged. Drop-out rates between arms did not reach significance (Fisher’s exact test = 0.21). The reasons for dropping out of the interventions included chronic health problems (*n* = 2) and being admitted to full-time school (*n* = 2). Three of the four families who dropped out post-allocation expressed disappointment with their allocation to SCIP-I.

Among the 19 participants who completed the trial, all but 2 participants completed 36 sessions. Two participants completed 31 sessions (one from each group). Sessions were typically delivered over a period between 4 and 6 months. Interruptions to the schedule of biweekly sessions were most commonly due to school vacations when parents chose not to undertake sessions. Children’s chronic illnesses and COVID infections within the families also impacted the frequency of sessions.

### Adverse events

As reported in the ‘Introduction’ section, music therapy has an established body of evidence for acceptability and safety for autistic children (e.g. Bieleninik et al., 2017). No specific adverse effects were documented in the wider literature (Stegemann et al., 2019). Nonetheless, our study aimed to establish feasibility of intervention delivery including the procedure of a RCT, hence we collected data on adverse events. In addition to adverse (i.e. concerns of risk or harm to the child and/or parent) and serious adverse events (i.e. clear evidence of immediate risk to welfare for the child and/or parent), commonly included in conventional clinical trials, we have devised another category labelled as ‘unintended effects’ (Junqueira et al., 2023). Unintended effects included clear evidence of distress, emotional or behavioural, in the child beyond their usual presentation and/or negative impact on their quality of life. Considering the clinical, language and behaviour profile of the study participants, a degree of behavioural disturbances and non-compliance with the intervention/study procedure was present throughout all sessions. We therefore collected data on unintended effects based on parents’ feedback using their intimate knowledge of their own child.

There were no safety concerns or adverse events reported during the trial. Among the sessions delivered across all participants, only three intervention sessions ceased because the child became distressed. Online vocabulary testing sessions were often ended due to child inattention or behaviour, in which cases parents would go through the tests with the therapist and explain what words their child could understand or speak at home.

### Outcome data

Table 3 shows the demographic information of the children in the MAP and SCIP-I groups. There were 5 female and 22 male participants with a mean age of 42.52 months (*SD* = 9.80) and a mean-measured VABS-3 intelligence quotient of 56.00 (*SD* = 4.60) and of whom 7 used echolalia. Among the 27 participants (13 MAP, 14 SCIP-I) who were randomised, 8 parent–child dyads (2 MAP, 6 SCIP-I) withdrew (see Supplementary Table 3 for full details), leaving 19 participants (11 MAP, 8 SCIP-I) who completed the trial and provided data for analysis (see Supplementary Table 2 for full details). Using minimisation randomisation, the two groups were balanced in terms of gender, developmental level and the presence/absence of echolalia.

**Table 3.** Demographic information of the MAP and SCIP-I groups.

Group	MAP (randomised)	SCIP-I (randomised)	MAP (trial)	SCIP-I (trial)
*N*	13	14	11	8
Gender	2 females; 11 males	3 females; 11 males	2 females; 9 males	2 females; 6 males
IQ (VABS-3)	55.46 (5.41)	56.50 (3.84)	56.18 (5.56)	55.88 (4.19)
Echolalia	3 yes; 10 no	4 yes; 10 no	2 yes; 9 no	1 yes; 7 no
Age (months)	43.31 (10.75)	41.79 (9.18)	40.36 (8.76)	38.50 (8.90)

Among the 27 participants (13 MAP, 14 SCIP-I) who were randomised, there were 8 withdrawals (2 MAP, 6 SCIP-I), leaving 19 participants (11 MAP, 8 SCIP-I) who completed the trial. IQ was estimated using VABS-3. The presence/absence of echolalia was based on parent reports. SCIP-I: Social Communication Intervention for Pre-schoolers–Intensive; MAP: music-assisted programmes; VABS-3: Vineland Adaptive Behaviour Scales–third edition; IQ: intelligence quotient.

The means and standard deviations of all outcome variables across different timepoints for the two groups are presented in Table 4, including adaptive functioning (VABS-3); social responsiveness (SRS-2); number of phrases understood, words produced and words understood (CDI); number of target words understood and produced (out of 36); receptive (ROWPVT-4) and expressive vocabulary (EOWPVT-4) and number of vocalisations, words, phrases, social responses and social initiations per minute (in parent–child interaction videos). Results from the mixed-effects models are presented in Table 5. There were no statistically significant main effects or interactions on adaptive functioning, number of target words understood and produced, receptive and expressive vocabulary, vocalisations, word production and phrase production during the videos. In the interest of space, we only report significant findings below.

**Table 4.** Means and standard deviations for the outcome measures of the MAP (*n* = 11) and SCIP-I (*n* = 8) groups.

Measure	Pre-intervention		Mid-intervention		Post-intervention		3-month follow-up	
	MAP	SCIP-I	MAP	SCIP-I	MAP	SCIP-I	MAP	SCIP-I
Adaptive functioning (VABS-3)	56.18 (5.56)	55.88 (4.19)	NA	NA	58.45 (5.47)	57.00 (4.96)	58.55 (6.35)	56.71 (6.55)
Autism severity (SRS-2)	79.00 (9.06)	79.13 (8.20)	NA	NA	74.91 (7.98)	81.25 (8.33)	74.82 (9.45)	78.86 (9.41)
Phrases understood (CDI)	10.27 (5.71)	7.88 (5.89)	11.36 (3.11)	9.38 (6.61)	15.55 (3.98)	12.00 (6.39)	14.91 (6.06)	13.86 (8.71)
Words produced (CDI)	1.45 (2.25)	1.63 (4.21)	9.18 (10.42)	7.63 (18.90)	10.36 (16.49)	13.25 (35.09)	27.18 (25.97)	27.00 (68.81)
Words understood (CDI)	62.00 (27.46)	66.63 (66.93)	93.55 (53.32)	94.13 (107.23)	106.27 (61.90)	108.00 (115.10)	136.82 (73.65)	128.14 (131.58)
Thirty-six target words understood	0.64 (1.80)	2.25 (6.36)	2.55 (6.86)	3.50 (9.90)	4.55 (9.73)	3.63 (10.25)	4.91 (10.28)	4.14 (9.32)
Thirty-six target words produced	0.00 (0.00)	0.00 (0.00)	0.64 (2.11)	3.25 (8.80)	0.09 (0.30)	0.50 (1.41)	2.36 (5.07)	4.29 (11.34)
ROWPVT	54.36 (0.50)	56.25 (3.96)	57.00 (9.95)	55.63 (3.85)	57.27 (10.85)	54.00 (0.00)	57.36 (6.67)	56.14 (5.67)
EOWPVT	55.27 (1.49)	55.63 (2.07)	54.55 (0.69)	54.63 (1.06)	54.36 (0.50)	54.43 (0.79)	55.18 (3.92)	57.86 (10.21)
Vocalisations per minute	3.04 (2.42)	4.08 (3.93)	3.05 (1.75)	7.05 (7.60)	4.51 (3.80)	4.92 (5.75)	4.33 (2.94)	3.45 (2.75)
Words per minute	0.13 (0.22)	0.18 (0.36)	0.27 (0.39)	1.17 (2.13)	0.87 (2.09)	0.55 (1.35)	1.13 (1.64)	1.01 (2.08)
Phrases per minute	0.00 (0.00)	0.00 (0.00)	0.62 (2.01)	0.01 (0.02)	0.16 (0.38)	0.00 (0.00)	0.56 (1.46)	0.04 (0.12)
Social responses per minute	1.07 (0.62)	1.18 (1.60)	NA	NA	NA	NA	1.55 (0.78)	1.05 (0.56)
Social initiations per minute	0.55 (0.42)	0.84 (0.52)	NA	NA	NA	NA	1.13 (0.71)	0.79 (0.73)

MAP: music-assisted programmes; SCIP-I: Social Communication Intervention for Pre-schoolers–Intensive; VABS-3: Vineland Adaptive Behaviour Scales–third edition; NA: not available; SRS-2: Social Responsiveness Scale–2; CDI: MacArthur-Bates Communicative Development Inventories; ROWPVT: Receptive One-Word Picture Vocabulary Test; EOWPVT: Expressive One-Word Picture Vocabulary Test.

**Table 5.** Results from the mixed-effects models (Type III analysis of variance table with Satterthwaite’s method) on the outcome measures.

Measure	Group	Timepoint	Group × timepoint
Adaptive functioning (VABS-3)	*F*(1, 17.14) = 0.29, *p* = 0.596	*F*(2, 33.24) = 1.73, *p* = 0.192	*F*(2, 33.24) = 0.36, *p* = 0.699
Autism severity (SRS-2)	*F*(1, 17.05) = 0.90, *p* = 0.357	*F*(2, 33.09) = 1.78, *p* = 0.184	*F* **(2, 33.09)** **=** **4.96, *p*** **=** 0.013*
Phrases understood (CDI)	*F*(1, 17.06) = 1.00, *p* = 0.331	*F* **(3, 50.11)** **=** **10.97, *p*** **<** 0.001***	*F*(3, 50.11) = 0.33, *p* = 0.800
Words produced (CDI)	*F*(1, 17.15) = 0.00, *p* = 0.990	*F* **(3, 50.26)** **=** **5.24, *p*** **=** 0.003**	*F*(3, 50.26) = 0.05, *p* = 0.986
Words understood (CDI)	*F*(1, 17.02) = 0.01, *p* = 0.941	*F* **(3, 50.04)** **=** **12.87, *p*** **<** 0.001***	*F*(3, 50.04) = 0.45, *p* = 0.721
Thirty-six target words understood	*F*(1, 17.04) = 0.00, *p* = 0.975	*F*(3, 50.07) = 2.46, *p* = 0.074	*F*(3, 50.07) = 0.68, *p* = 0.571
Thirty-six target words produced	*F*(1, 17.20) = 0.53, *p* = 0.478	*F*(3, 50.36) = 2.67, *p* = 0.057	*F*(3, 50.36) = 0.44, *p* = 0.723
ROWPVT	*F*(1, 17.28) = 0.18, *p* = 0.677	*F*(3, 49.52) = 0.37, *p* = 0.776	*F*(3, 49.52) = 1.12, *p* = 0.350
EOWPVT	*F*(1, 17.40) = 0.86, *p* = 0.366	*F*(3, 50.35) = 1.27, *p* = 0.295	*F*(3, 50.35) = 0.54, *p* = 0.659
Vocalisations per minute	*F*(1, 17.21) = 0.57, *p* = 0.462	*F*(3, 49.41) = 1.12, *p* = 0.352	*F*(3, 49.41) = 2.60, *p* = 0.062
Words per minute	*F*(1, 17.16) = 0.05, *p* = 0.832	*F*(3, 49.37) = 2.58, *p* = 0.064	*F*(3, 49.37) = 1.32, *p* = 0.279
Phrases per minute	*F*(1, 17.45) = 1.72, *p* = 0.206	*F*(3, 50.23) = 0.50, *p* = 0.682	*F*(3, 50.23) = 0.43, *p* = 0.735
Social responses per minute	*F*(1, 17) = 0.31, *p* = 0.587	*F*(1, 17) = 0.45, *p* = 0.514	*F*(1, 17) = 1.43, *p* = 0.249
Social initiations per minute	*F*(1, 17) = 0.01, *p* = 0.922	*F*(1, 17) = 3.48, *p* = 0.079	*F* **(1, 17)** **=** **5.26, *p*** **=** 0.035*

VABS-3: Vineland Adaptive Behaviour Scales–third edition; SRS-2: Social Responsiveness Scale–2; CDI: MacArthur-Bates Communicative Development Inventoriesy; ROWPVT: Receptive One-Word Picture Vocabulary Test; EOWPVT: Expressive One-Word Picture Vocabulary Test.

* *p* < 0.05; **p < 0.01; ***p < 0.001.

**Figure 2.**
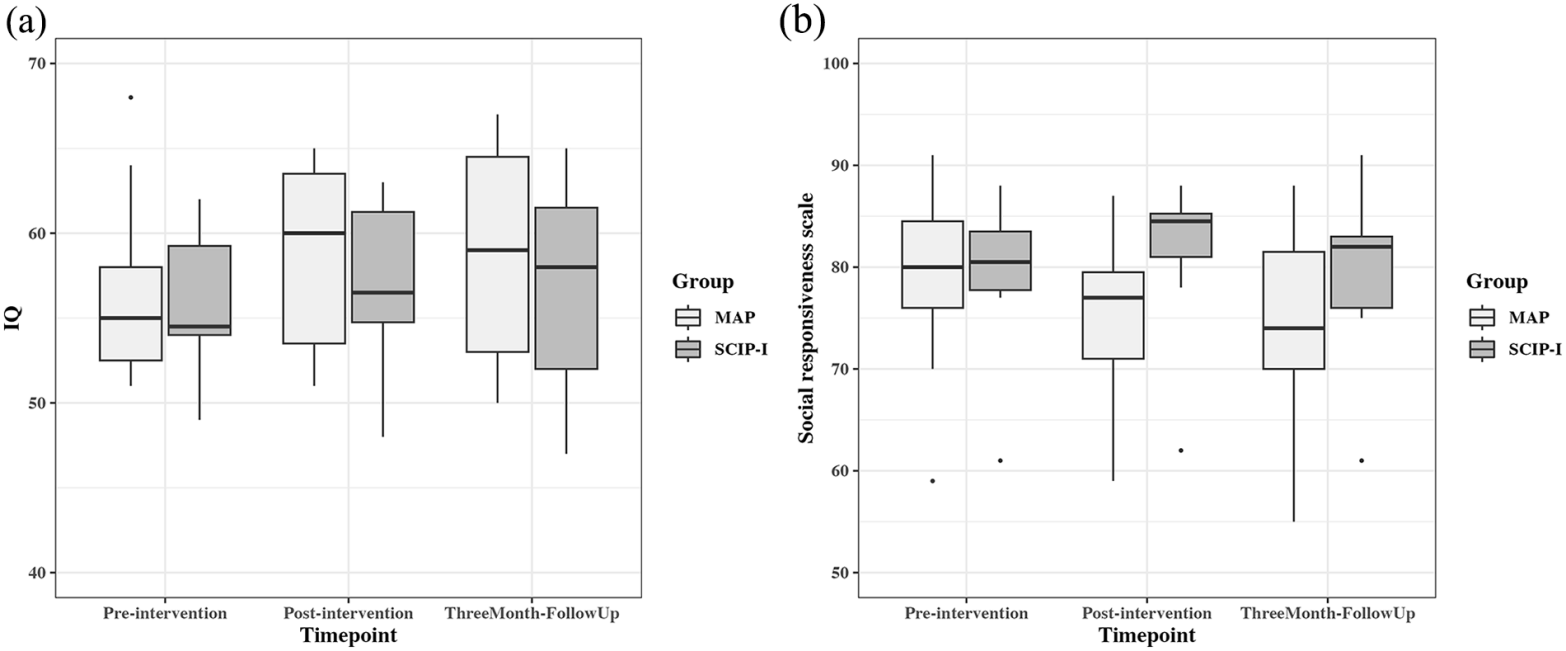
Adaptive behaviour composite scores (VABS-3) and social responsiveness scale (SRS-2) scores for the MAP group and the SCIP-I group across three timepoints. (a) IQ (adaptive behaviour composite on VABS-3) and (b) Social responsiveness scale on SRS-2.

Figure 2 shows the boxplots of the two groups’ scores on the VABS-3 and SRS-2 across three timepoints. The data from the adaptive behaviour composite scores on the VABS-3 (Figure 2(a)) showed no statistically significant effects. The SRS-2 data (see Figure 2(b)) show that there was a group × timepoint interaction (*F*(2, 33.09) = 4.96, *p* = 0.013), suggesting that the MAP group increased social responsiveness more over time than those receiving SCIP-I. Post hoc pairwise analysis using the Kenward–Roger method for degrees of freedom identified that the increased social responsiveness in the MAP group was evident in the comparisons between baseline and post-intervention (mean difference = 4.09, *t*(33) = 3.11, *p* = 0.010) and between baseline and 3-month follow-up (mean difference = 4.18, *t*(33) = 3.18, *p* = 0.009).

**Figure 3.**
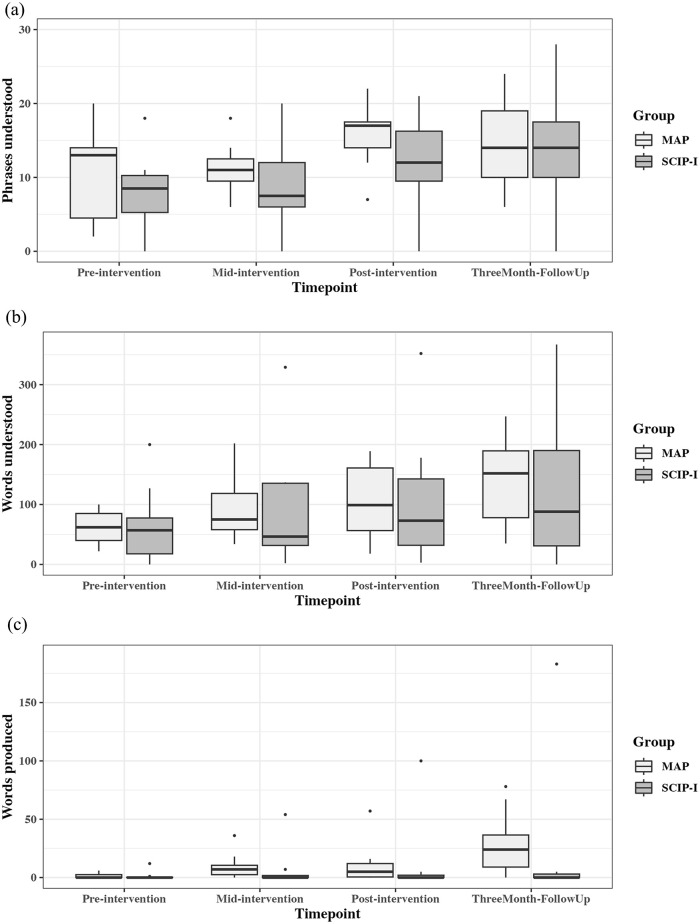
Numbers of phrases understood, words understood and words produced on the CDI by the two groups across four timepoints. (a) Phrases understood on CDI; (b) words understood on CDI; and (c) words produced on CDI.

The results from the CDI data are shown in Figure 3. There was a significant main effect of timepoint on phrases understood (*F*(3, 50.11) = 10.97, *p* < 0.001), words produced (*F*(3, 50.26) = 5.24, *p* = 0.003) and words understood (*F*(3, 50.04) = 12.87, *p* < 0.001). Subsequent pairwise comparisons showed that the results were due to more phrases being understood by the MAP group between baseline and post-intervention (difference = 5.27, *t*(50) = 3.84, *p* = 0.002), baseline and 3-month follow-up (difference = 4.64, *t*(50) = 3.37, *p* = 0.008) and mid-intervention to post-intervention (difference = 4.18, *t*(50) = 3.04, *p* = 0.019). The SCIP-I group showed only one significant increase in number of phrases understood (baseline to 3-month follow-up; difference = 5.56, *t*(50.3) = 3.30, *p* = 0.009). Similar pairwise comparisons for words produced found that only the baseline to 3-month follow-up for the MAP group was statistically significant (difference = 25.73, *t*(50) = 3.12, *p* = 0.015). The pairwise comparisons for the words understood found that the number increased for the MAP group between baseline and post-intervention (difference = 44.3, *t*(50) = 3.31, *p* = 0.009), baseline and 3-month follow-up (difference = 74.8, *t*(50) = 5.60, *p* < 0.001) and mid-intervention to 3-month follow-up (difference = 43.3, *t*(50) = 3.24, *p* = 0.011). The SCIP-I group showed only one significant increase in number of words understood (baseline to 3-month follow-up; difference = 52.6, *t*(50.1) = 3.21, *p* = 0.012).

**Figure 4.**
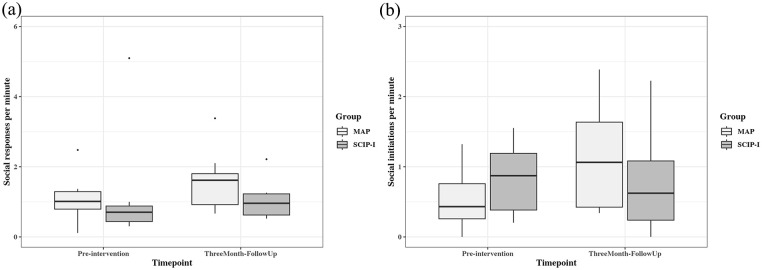
Numbers of social responses and social initiations per minute by the two groups across two timepoints in parent–child interaction videos. (a) Social responses per minute in parent–child interaction videos and (b) social initiations per minute in parent–child interaction videos.

Analysis of the video recordings on social communication is shown in Figure 4. A significant group × timepoint interaction was observed for social initiations only (Figure 4(b); *F*(1, 17) = 5.26, *p* = 0.035). Post hoc analysis showed that social initiations by the child were more frequent in the MAP group (difference from baseline = 0.58, *t*(17) = 3.21, *p* = 0.005) than in the SCIP-I group (difference from baseline = 0.06, *t*(17) = 0.28, *p* = 0.782) at 3-month follow-up. There were no statistically significant findings in the analysis of social responses of the child (Figure 4(a)).

Finally, based on video recordings of parent–child interactions and clinical observations of the therapist, four of the MAP group and one of the SCIP-I group reached Phase 3 of spoken-language development – word combinations.

## Thematic analysis of parents’ perceived acceptability of MAP

Six parents who completed MAP were approached, and mothers of five boys consented to interviews (Table 6). Interviews lasted 44 min on average. The main themes emerging from analysis of the parent interviews were (1) parents’ learning through the intervention process, (2) key components of MAP and (3) views about personalised treatment.

**Table 6.** Characteristics of the parents (and their child) who took part in the interviews.

Parent number	Parent	Age of child at interview (years)	Gender of child	Child’s diagnosis
Parent 1 (P1)	Mother	5;1	Male	Autism
Parent 2 (P2)	Mother	3;3	Male	Autism and GDD
Parent 3 (P3)	Mother	4;0	Male	Autism
Parent 4 (P4)	Mother	5;5	Male	Autism and GDD
Parent 5 (P5)	Mother	3;8	Male	Autism and ARFID

All children completed 36 sessions. GDD: global developmental delay; ARFID: avoidant restrictive food intake disorder.

Parents frequently mentioned the need to fully commit to the programme for the best outcomes. Parents came to understand their role as agents of intervention and reflected on their experience that some strategies were easier to learn and integrate into everyday life than others (e.g. following child’s lead) and that some strategies had more impact than others (e.g. providing time for the child to respond). Parents increased understanding of their child’s strengths and needs (e.g. appreciating existing and emerging communication skills). Parents learnt that their child’s improvements spanned across quality of interactions (P5) and quantity of social interactions (P3), as highlighted below:

He was more present . . . before MAP he would never involve in any game with me. Like he, he was just playing by himself, he would sit backwards to me if I was trying to get involved . . . But during MAP, he started actually playing with me. (P5)

. . . as soon as I put these particular songs on, he will come and say ‘up’ and we’ll dance in the kitchen and he loves doing that. He’s never done that before. (P3)

Parents recognised how structural components of MAP worked together and how the therapist facilitated progress. The requirement to record, upload and review videos was time-consuming, but was noted as essential to learning. Accessing and engaging in songs and strategies were identified as key ingredients of MAP. Parents understood that detailed information gathering was required through assessments and questionnaires, and these carried benefits (e.g. opportunity to see progress over time) and drawbacks (e.g. highlighting reality of child’s needs and burden of completion). Parents also understood that intervention was delivered online which afforded advantages (e.g. not breaking child’s routine) and disadvantages (e.g. therapist not gaining full picture of play space).

Parents frequently spoke about their preference for personalised treatment, matching their child’s interests and progress. MAP songs were frequently reported to capture children’s attention and many children had song preferences; some songs were still sung post intervention. However, reservations were raised by some parents regarding (1) the pre-selected target words (i.e. when asked whether target words were important, one parent states ‘Not necessarily those particular words’ (P1)), (2) the number of songs (i.e. one parent mentions ‘There was quite a lot of them . . . so maybe, well, for him, like maybe fewer . . ., it was quite a high turnover of different songs’ (P4)) and (3) the routine provision of pictures to accompany songs (i.e. one parent highlighted ‘I mean, in some of the sessions we were forced to have pictures in front of him, although he was not very keen on that’ (P2)). Parents valued how the therapist delivered MAP as specified while personalising it to individual families’ situations. Parents suggested adding flexibility to the MAP format (e.g. using pictures if a device was distracting) and individualised therapy goals (e.g. aiming to increase understanding rather than verbalisation for non-verbal children).

## Discussion

This trial demonstrated that it was possible to identify, recruit and retain young autistic children with minimal verbal language (see the ‘Introduction’ for definition) for an RCT comparing MAP with SCIP-I. The intervention proved safe to deliver and was acceptable to the parents and children. The feasibility of the trial was also evidenced by the low dropout rate after the first intervention session. The reasons for post-randomisation dropout from the trial included persistent illness of child or caregiver, during a global pandemic. There were no adverse incidents over the course of the trial. A particular feature of this trial was the online delivery and the use of an active control group. Interviews with the parents in the MAP arm found that music was motivating for the children. Using a smartphone app to aid the intervention proved helpful for providing stimuli and musical contexts for communication.

## Strengths and weaknesses of the study

One major strength of this trial is that it compared two active treatments with comparable retention of participants. This offers significant advantages over trials which compare a novel intervention with a control arm receiving routine care (Aldred et al., 2004), treatment as usual provided by local hospital and community services (Green et al., 2010, 2022), a control condition (Boyd et al., 2014) or on a waitlist (Heitzman-Powell et al., 2023). Furthermore, our approach ensured that the experimental and control children did not differ in intervention dose received during the trial.

There were no adverse events recorded and dropout seemed to have been due to ill health of either the child or the parent. The demands on parents were perceived as acceptable. Although the trial was not powered to provide evidence of a difference, the differential outcomes between interventions suggest that a larger trial is warranted particularly as both arms used active interventions. From a parental point of view, the remote method of assessment and intervening with them and their children reduces the costs of travel and time at the clinic.

There were some difficulties revealed by the research. Some of the measures used could not be delivered satisfactorily online or on video. This applies particularly to standardised assessments usually delivered face to face by trained personnel (e.g. EOWPVT-4). In a future trial, we would intend to carry out some assessments in a clinic setting or in the participants’ homes. Technical issues meant that measuring parent–child interactions using videos self-recorded by parents proved problematic. This may require the presence of a research assistant in the home to ensure representativeness and validity of the video recording and to ensure correct delivery of some assessments. An additional component to the app might be including functions for recording what parents do with their children and what they continue to do following the trial. If the app included a method of video recording caregiver–child intervention sessions, it could also be useful for those therapists working with families who are unable to attend clinic sessions. A further limitation is that out of a possible 13 MAP parents, only 5 whose children completed the trial took part in the interview study. Due to the self-selection nature of the sample, these parents’ views may not give a full picture of the acceptability of the MAP intervention. Apart from the remaining eight MAP parents, the viewpoints of those in the SCIP-I arm were also not gathered.

## Potential underlying mechanisms

The results of this study are in line with those of the PACT (Preschool Autism Communication Trial) trial of parent-mediated social communication therapy for young children with autism (Green et al., 2010) in that the largest changes are seen in social interaction rather than in language development. In itself, this is encouraging since language development is rooted in the social interactions of parent and child (Levickis et al., 2022). The mechanisms underlying the improvements in social interactions through use of songs/music are likely associated with the biological and cultural functions of music on social bonding (Savage et al., 2021), which in turn increases social communication skills such as imitation and joint attention (Kraus & White-Schwoch, 2016). These skills are vital in developing language in autistic and non-autistic children (Luyster et al., 2008). A recent intervention study suggests that music improves social communication and functional brain connectivity between auditory and fronto-motor regions in autism (Sharda et al., 2018). Evidence also suggests that improvements in parents’ responsiveness to their child’s behaviour are an active ingredient of parent-mediated early interventions (Gulsrud et al., 2016; Pickles et al., 2015). Thus, music may provide a mechanism for both accelerating the enhancement of parental synchrony associated with standard parent-mediated interventions and increasing the child’s social communication and language skills. The therapeutic music capacities model proposes seven properties of music that induce cognitive, psychosocial, behavioural and motor benefits for autistic individuals (Brancatisano et al., 2020). Thus, music-based language interventions have the potential to open the neural pathways to language in the autistic brain and to create genuine and lasting outcomes to address the socio-neuro-constructionist issues in autism (López, 2015).

## Implications for future research

The total loss of possible participants from initial enquiry (*n* = 72) to completing the trial amounted to 79% of the potential sample (*n* = 91). The main reason was that most of the children lacked an official diagnosis of autism given the long waiting list for autism assessment in the United Kingdom (Crane et al., 2016; Russell et al., 2022), thus not fulfilling our inclusion criteria. A future trial should be planned to allow for only one fifth of potential participants completing the trial, suggesting that any future trial should plan on being able to interest almost five times as many people as will complete. The design of the full-scale trial would need to allow for about 70% dropout before randomisation and 30% dropout post randomisation, or recruit through more targeted routes, for example screening autism clinic caseload. A future trial should incorporate more idiosyncratic vocabulary goals and preferred songs as requested by parents.

The data on word combinations were used to calculate the sample size needed for an efficacy trial based on the power to detect an odds ratio of 5.7 at a power of 90% and 0.05 alpha. This suggests that at least 58 children per arm will be required. Inflating for a predicted loss to follow-up of 20% gives a total target recruitment sample of 146. This sample size will be large enough to meet larger scale feasibility objectives, carry out exploratory analyses and define more accurate parameters for a definitive RCT.

In conclusion, this trial suggests that it is feasible to run a trial of online MAP for initial word learning in preschool autistic children. Training parents to deliver social communication therapy is an effective way to improve social responsiveness and spoken language in autistic children and is recommended by the NICE guideline (National Institute for Health and Clinical Excellence, 2013). The intervention could be supported by the development of a widely available smartphone app to record and review homework sessions and provide the musical basis for the intervention. Future trial should further incorporate parental feedback and draw on parental experiences to inform an ecologically valid and effective intervention.

## Supplemental Material

sj-docx-1-aut-10.1177_13623613241233804 – Supplemental material for Using music to assist language learning in autistic children with minimal verbal language: The MAP feasibility RCT Supplemental material, sj-docx-1-aut-10.1177_13623613241233804 for Using music to assist language learning in autistic children with minimal verbal language: The MAP feasibility RCT by Tim I Williams, Tom Loucas, Jacqueline Sin, Mirjana Jeremic, Sina Meyer, Sam Boseley, Sara Fincham-Majumdar, Georgia Aslett, Ruan Renshaw and Fang Liu in Autism
