# Supplementary material for: Using music to assist language learning in autistic children with minimal verbal language: The MAP feasibility RCT
Source: Autism. 2024 Mar 3;28(10):2515–33. doi: 10.1177/13623613241233804 (PMC11504703; doi:10.1177/13623613241233804)
Supplement: sj-docx-1-aut-10.1177_13623613241233804 – Supplemental material for Using music to assist language learning in autistic children with minimal verbal language: The MAP feasibility RCT [file sj-docx-1-aut-10.1177_13623613241233804.docx]

**Supplementary Material**

**Using music to assist language learning in autistic children with minimal verbal language: The MAP feasibility RCT trial**

**Supplementary Table 1**. MAP songs.

| Song 1: Hello song  **Hello, mummy**. Hello, mummy, how’re you  Hello **daddy,** hello daddy, how’re you  Hello …. Hello…. How’re you?  Hello …. Hello…. How’re you?  Hello, mummy. Hello, mummy, how’re you  Hello daddy, hello daddy, how’re you  Hello …. Hello…. How’re you?  **Hugs** and **kisses** all around  We’ll have so much fun today  Singing, dancing and we’ll play  Let’s get started everyone  Play some music and have fun! | Song 2: Bye-bye song  **Bye-bye** bye-bye everyone  I hope you have all had fun  We had so much fun today  Singing, dancing and we played  Bye-bye bye-bye everyone  Hugs and kisses all around  We will see you very soon  To play some music all **again!** |
| --- | --- |
| Song 3: Play inside song  In my **home**  There is lots to do  What will I play with first?  I can **play** with my **toys, book, tv** | Song 4: Play outside song  Outside**, outside,**  All day long  Outside, outside  And play all day  Outside, outside,  What is there today?  We can **go** to the **park**  We can **play** with the **ball**  We can play with the **dog**  We can play with the **cat** |
| Song 5: Night-night song  **Bath-tim**e, bath-time  its almost bath time  Lot’s of **bubbles**  Can we **pop** them?  Bath-time you were lots of fun  But we are **finished** now  Night-time, night-time  it’s almost night time  The **bed** is ready  Time for **sleeping**  **Shhhhhh** | Song 6: Fall over song  Bumpty-bumpty bump  I fall over  **Uh-oh,** uh-oh  what will we do  Bumpty-bumpty bump  I fall over  Uh-oh, uh-oh  **Ouch** ouch ouch  Bumpty-bumpty bump  I fall over  Uh-oh, uh-oh  **Help** help help!!  What’s is wrong?  I fall over  Uh-oh, uh-oh  How can I help? |
| Song 7: Food song  Would you like some **food**?  **Yes** or **no**  Would you like some **drink?**  No No No  Would you like some ….  Yes or no  Would you Like some **more**?  Please please **please**  There you go  **Thankyou**  Yummy Yummy **Yummy** | Song 8: Colours song  A rainbows in the sky today with all of its colours  Look, **look**!  What colours can you see?  **Red, green, blue, yellow**  What else can be red? |
| Song 9: Feelings song  **Happy** happy happy  I am happy  And when I’m happy I clap my hands  **Hungry** hungry hungry  I am hungry  And when I’m hungry I rub my belly  **Tired** tired tired  I am tired  And when I’m tired do a stretch  **Sad** sad sad  I am sad  And when I’m sad I rub my eyes | Song 10: Cat song  I see a cat sitting over there  Uh-oh **uh-oh**  he looks **hungry**  Let’s give him some **food**  Yummy **yummy**  The cat is smiling  I think he’s **happy**  I see a cat sitting over there  Uh-oh uh-oh  he looks **sad**  Let’s give him some toys  **Yay**  The cat is smiling  I think he’s happy  I see a cat sitting over there  Uh-oh uh-oh  he looks **tired**  Let’s put him to **bed**  **Shhhhh** |
| Song 11: Traffic light song  The **car** goes brum brum  Down the road  The car goes brum brum  All day long  **Stop!** **Look!**  The light is **red**  The car goes brum brum  Down the road  The car goes brum brum  All day long  Stop! Look!  The light is **yellow**  The car goes brum brum  Down the road  The car goes brum brum  All day long  Go! Look!  The light is **green**  No I can carry on driving |  |

**Supplementary Table 2**. Data collected during the trial.

| Data collected for the 19 participants who completed the trial (11 MAP and 8 SCIP-I):   - **36 target words** collected at four time points: before the intervention (all), 12 weeks into the intervention (all), post intervention (all), three months follow up (all MAP and 7 SCIP-I with 017 missing) - **CDI** collected at four time points: before the intervention (all), 12 weeks into the intervention (all), post intervention (all), three months follow up (all) - **ROWPVT** collected at four time points: before the intervention (all), 12 weeks into the intervention (all), post intervention (all), three months follow up (all MAP and 7 SCIP-I with 017 missing) - **EOWPVT** collected at four time points: before the intervention (all), 12 weeks into the intervention (all), post intervention (all), three months follow up (all MAP and 7 SCIP-I (017)) - **SRS** collected at three time points: before the intervention (all), post intervention (all), three months follow up (all MAP and 7 SCIP-I with 017 missing) - **VABS** collected at three time points: before the intervention (all), post intervention (all), three months follow up (all MAP and 7 SCIP-I with 017 missing) - **Videos**collected at four time points: before the intervention (all), 12 weeks into the intervention (all), post intervention (all), three months follow up (10 MAP with 005 missing and 7 SCIP-I with 017 missing) |
| --- |
| Data collected for the randomised participants who did not complete the trial (2 MAP and 6 SCIP-I):   - **002**: CDI, SRS, VABS, ROWPVT, EOWPVT - **006**: CDI, SRS, VABS, ROWPVT, EOWPVT - **007**: all baseline assessments - **012**: CDI, SRS, VABS - **015**: CDI, SRS, VABS, ROWPVT, EOWPVT - **019**: all baseline assessments - **022**: all baseline assessments - **024**: CDI, SRS, VABS, ROWPVT, EOWPVT |

**Supplementary Table 3**. Supplementary information for CONSORT diagram.


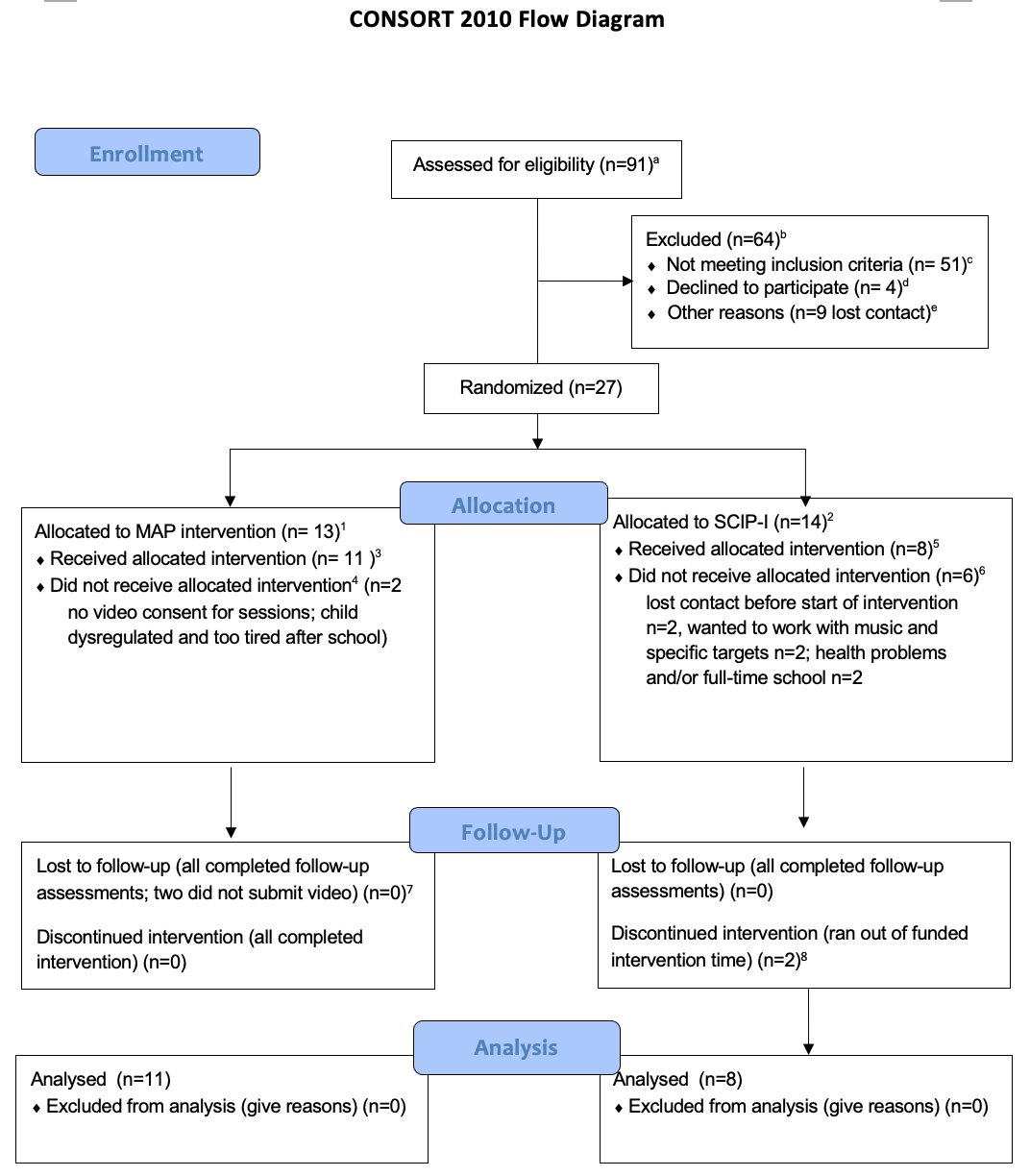


| ^a^ we have been contacted via registration form link (64), email (19), phone (6), word of mouth (2); assessed for eligibility mainly means through initial interview when gathering information about the child’s diagnosis, language, age; not all these participants will have been sent VABS, SRS and CDI. |
| --- |
| ^b^/^c^ children mainly too verbal or over 5 years old; this would become apparent in the initial interview or once parents filled in CDIs or during direct language assessments. |
| ^d^ when declining to participate parents expressed concerns over the length of intervention, tele-health, juggling 18 weeks of intervention with work and school commitments. |
| ^e^ lost contact. |
| ^1^ 001, 004, 005, 008, 012, 013, 014, 016, 018, 022, 023, 026, 027 = 13 |
| ^2^ 002, 003, 006, 007, 009, 010, 011, 015, 017, 019, 020, 021, 024, 025 (028 is 025) = 14 |
| ^3^ 001, 004, 005, 008, 013, 014, 016, 018, 023, 026, 027 = 11 |
| ^4^ 012 (withdrew video consent) 022 (attrition following 6 weeks of intervention as child with sensory and sleeping issues and difficult to coordinate with full-time school hours – child too tired after school) = 2 |
| ^5^ 003, 009, 010, 011, 017 (completed 31 sessions – child having repeated ear infections; complex family situation including parents separating), 020 (completed 31 sessions; multiple covid infections in the family and having to self-isolate on multiple occasions), 021, 025 (in data 028) = 8 |
| ^6^ 002 (screened before summer; by the time we were able to offer intervention in mid-September 2020, the parent had found a private SLT with whom she wanted to work instead of joining the intervention), 006 (parent disappointed the child was not allocated to the music group and felt the child was too able for the intervention then stopped answering emails and calls), 007 (mother and child hospitalised, mother became pregnant and felt child too tired to continue with the sessions after school so withdrew after 7 weeks of intervention), 015 (completed all the parent reported tests and language tests; complex family situation with two disabled children), 019 (completed 6 weeks of intervention, withdrew after child started full-time school as too tired after school hours), 024 (completed all tests but withdrew as parent wanted to be in the music arm and wanted to work on specific targets) = 6 |
| ^7^005 and 023 did not complete the follow-up ten minute parent-child interaction video |
| ^8^017 (completed 31 sessions – child having repeated ear infections; complex family situation including parents separating), 020 (completed 31 sessions; multiple covid infections in the family and having to self-isolate on multiple occasions) |
